# Progression of *Coxiella burnetii* infection after implementing a two-year vaccination program in a naturally infected dairy cattle herd

**DOI:** 10.1186/s13028-014-0047-1

**Published:** 2014-07-22

**Authors:** Alvaro Piñero, Jesús F Barandika, Ana Hurtado, Ana L García-Pérez

**Affiliations:** 1 Departamento de Sanidad Animal, NEIKER- Instituto Vasco de Investigación y Desarrollo Agrario, Berreaga 1, Derio, 48160, Bizkaia, Spain

**Keywords:** Coxiella burnetii, Cattle, Vaccination, Environment, Control

## Abstract

**Background:**

The high prevalence of *Coxiella burnetii* infection in dairy cattle herds recently reported and the long survival time of the bacterium in the environment pose a risk to human and animal health that calls for the implementation of control measures at herd level. This study presents the results of a 2-year vaccination program with an inactivated phase I vaccine in a Spanish dairy herd naturally infected with *C. burnetii*. Calves older than 3 months and non-pregnant heifers and cows were vaccinated in April 2011 and the farm was subsequently visited at a monthly basis for vaccination of recently calved cows and calves that reached the age of 3 months. Annual booster doses were given to previous vaccinated animals as well. The effectiveness of the vaccine was assessed in terms of level of *C. burnetii* shedding through milk and uterine fluids and environmental contamination as determined by polymerase chain reaction (PCR).

**Results:**

The percentage of shedder animals through uterine fluids and milk progressively decreased, and *C. burnetii* DNA load in bulk-tank milk samples was low at the end of the study. The average seroconversion rate in not yet vaccinated animals, which acted as control group, was 8.6% during the first year and 0% in the second year. DNA of *C. burnetii* was found in aerosols and dust samples taken in the calving area only at the beginning of the study, whereas slurry samples remained *C. burnetii* PCR positive for at least 18 months. Multiple Locus Variable number tandem-repeat Analysis identified the same genotype in all *C. burnetii* DNA positive samples.

**Conclusions:**

In the absence of any changes in biosecurity, the overall reduction of *C. burnetii* infection in animals to 1.2% milk shedders and the reduced environment contamination found at the end of the study was ascribed to the effects of vaccination together with the culling of milk shedders. Vaccination has to be planned as a medium-long term strategy to suppress risks of re-infection.

## Background

*Coxiella burnetii* is the causative agent of Q fever, a zoonotic disease considered an emerging public health problem, especially after the outbreak in the Netherlands, where more than 4,000 human cases have been notified since 2007 [[1],[2]]. A broad range of animal species have been identified as reservoirs of *C. burnetii*, though domestic ruminants are considered the most important source of infection for humans [[3]]. Infected animals shed bacteria through milk, faeces, vaginal fluids and birth products [[4],[5]] but milk is the main excretion route in cattle [[5]]. Inhalation of aerosols contaminated with extracellular forms of *C. burnetii* shed by infected animals is the main route of infection for humans and also for non-immune animals, especially when environmental conditions are favorable for the spread of bacteria [[6],[7]].

The high prevalence of *C. burnetii* infection in dairy herds reported in recent studies [[8],[9]] and the long survival capacity of this bacterium in the environment [[10]] call for the implementation of control measures aimed at reducing the exposure level at herd level. Control measures based on treatment with antibiotics or vaccination have been implemented. A recent study reported that antibiotics administered to dairy cattle at the drying-off period significantly prevented *C. burnetii* shedding around calving [[11]]. However, once infection is established in a herd, antibiotics are not able to reduce bacterial load shed by infected animals [[11],[12]]. So when infection is established in herds and animal shedders are contaminating the environment through feces or vaginal excretions, the implementation of a vaccination program is necessary to protect susceptible animals from being infected. Vaccine composition (*C. burnetii* in phase I or virulent, with complete lipopolysaccharide (LPS) and *C. burnetii* in phase II or not virulent, with incomplete LPS) determines their effectiveness with vaccines with phase I *C. burnetii* being more effective than those using phase II bacteria [[13],[14]]. Previous studies reported the effectiveness of vaccination in reducing the probability of a susceptible animal becoming a shedder [[15]] and the *C. burnetii* shedding level both in experimental and natural infections in sheep [[16]], goats [[13]] and dairy cattle [[11],[17]]. Taking into account the overall benefits of vaccination, the aim of this study was to monitor the progression of *C. burnetii* infection in a naturally infected commercial dairy cattle herd along 2 years of vaccination and culling of milk shedders by measuring *C. burnetii* DNA levels in the environment (air and dust from animal premises and slurry samples) and progression of bacterial shedding in animals.

## Methods

### Selected herd

At the end of 2010, *C. burnetii* infection was diagnosed in a dairy cattle herd (n = 252) with an abortion rate of 4%. Fetuses and placentas were not available for laboratorial analyses but presence of *C. burnetii* DNA was confirmed in vaginal mucus from aborted and calving cows (9/11 vaginal swabs) by polymerase chain reaction (PCR). Enzyme-linked immunosorbent assay (ELISA) was performed on sera of 17 cows and seroprevalence against *C. burnetii* was determined to be 23.5% (4/17). The distribution of *C. burnetii* was further investigated based on these preliminary data. Hence, a bulk-tank milk (BTM) sample, blood (sera) and faeces from all the animals in the herd and individual milk from all lactating cows were collected (March 2011) and analyzed by individual ELISA and/or PCR. Animals were classified into two categories “infected” and “non-infected”. Individuals with antibodies against *C. burnetii* and/or being PCR positive were considered “infected” and otherwise “non-infected”. A seroprevalence of 40% was found in first calving cows, and 9% of animals shed *C. burnetii* through milk and 0.4% through faeces. According to the EFSA criteria [[18]], presence of *C. burnetii* DNA in vaginal mucus of aborting cows and a herd seroprevalence of around 50% are indicative of active *C. burnetii* infection. Therefore, and in agreement with the farmer, a 2-year culling and vaccination plan was prepared and implemented from April 2011 onwards. Biosecurity level was not changed during this period and herd management continued as usual.

Spanish ethical guidelines (RD 1201/2005) and animal welfare regulations were strictly respected. Experimental work was officially approved by competent local authorities on health and animal welfare (Bizkaiko Foru Aldundia, Reference 10559, 3^rd^ November 2010).

### Vaccination strategy

To obtain optimal results from vaccination, recommendations derived from a previous vaccination study [[15]] were followed and the vaccine was applied to calves older than three months of age and non-pregnant heifers and cows. Individual data about age and reproductive status were compiled for all animals. Thus, vaccination started in April 2011 and, according to the manufacturer's instructions, each animal was given 2 doses 3 weeks apart of 4 ml of inactivated phase I vaccine (Coxevac, CEVA Santé Animale, Libourne, France) subcutaneously in the neck area using sterile single-use needles and syringes. Each 4 ml vaccine dose contained purified phase I *C. burnetii* corpuscular antigens (100 μg/ml) inactivated by formaldehyde. After this initial vaccination in April 2011, the farm was visited at a monthly basis, and all newly incorporated three month-old female calves, heifers that reached age for their first artificial insemination and all cows that calved within that month, received their first dose of vaccine followed by the second dose 3 weeks later. The target was to vaccinate all the animals in the herd within one year. Annual booster doses were given to all the animals before artificial insemination. Bull calves, which were removed from their dams after colostrum intake and fed on artificial milk until being sold at 4 months of age, were not vaccinated.

Considering that Q fever is a zoonosis, no control group of non-vaccinated animals was left in the herd, and all animals were vaccinated according to the protocol. Instead animals not yet vaccinated according to the protocol, e.g. cows being pregnant at the beginning at the study, served as controls until they were vaccinated.

### Sampling strategy

Serum samples were taken from animals prior to vaccination at the monthly visits to the farm along the first year (April 2011-March 2012) to compare serological results (presence/absence of antibodies against *C. burnetii*) with previous results obtained in March 2011 (seroconversion rate). Similarly, in April 2012 sera were taken from all the animals in the herd to evaluate seroconversions occurring in not yet vaccinated animals during the second year of the study (April 2012-March 2013). *C. burnetii* shedding in recently calved cows was assessed by PCR of uterine fluid samples taken immediately after calving.

To assess the evolution of *C. burnetii* shedding through milk, BTM samples were collected monthly and individual milk samples were collected from all milking cows every 6 months to evaluate changes in the percentage of animal shedders.

To determine environmental contamination with *C. burnetii*, samples collected included slurry sampled at a monthly basis, air (aerosols) sampled every 6 months using a Sartorius air sampler (Air Sampler, MD8 airscan, Goettingen, Germany) at a flow rate of 100 l/min for 10 min and collecting particles in gelatine filters, and dust collected from different animal premises surfaces (swabs) every 6 months. Air and dust samples were collected from the calving, breeding, and milking cows’ resting areas. In each sampling, 2 air samples and a maximum of 10 dust samples were collected per area.

### Laboratory analyses

#### Serological analyses

BTM, individual milk and serum samples were tested for the presence of antibodies against *C. burnetii* using a commercial indirect ELISA according to manufacturer’s instructions (LSIVET Ruminant Milk/Serum Q Fever kit; Laboratoire Service International, Lissieu, France). The antigen used was isolated from domestic ruminants at INRA, Nouzilly (France). A cocktail of antigen phases I and II was used in this assay to detect total anti-*C. burnetii* immunoglobulin G antibodies (IgG). The sample-to-positive control (S/P) ratio was calculated as follows: S/P = (OD sample – OD NC)/(OD PC – OD NC), where OD sample = optical density of the sample, OD NC = optical density of the negative control, and OD PC = optical density of the positive control. The results were expressed as an index: S/P × 100.

In the case of serum samples, S/P indices ≤40 were considered negative, whereas indices >40 were indicative of positive serum. On the other hand, milk samples with S/P indices ≤30 were considered negative, whereas samples with indices >30 were considered positive for *C. burnetii* antibodies.

#### PCR/qPCR analyses

Vaginal swabs, BTM samples, individual milk samples, air (gelatine filters) and dust samples were subjected to DNA extraction using the QIAmp DNA Blood Mini Kit (Qiagen, Valencia, CA, USA) as previously described [[19],[20]]. Slurry samples were treated according to a protocol adding 1 ml Phosphate Buffer Saline (Ambion, Life Technologies, Alcobendas, Madrid, Spain) to 0.3 g slurry, then vortexed for 3 min and centrifuged at 100 *g* for 1 min. Finally, 175 μl of supernatant were subjected to DNA extraction using the MagMax Total Nucleic Acid Isolation Kit (Ambion) following the manufacturer’s instructions.

A conventional PCR was carried out using primers targeting a transposon-like IS*1111* repetitive region of *C. burnetii* as described elsewhere [[21],[22]] adding a maximum of 70 ng of DNA template to each reaction. Negative controls were included every ten samples to rule out DNA contamination. After a PCR positive result was confirmed, the bacterial burden was quantified by quantitative real-time PCR (qPCR) using primers previously described [[23]] that target the IS*1111* region, and adding a commercially available TaqMan® Exogenous Internal Positive Control (Applied Biosystems, Foster City, CA, USA). Reactions were carried out using an ABI 7500 FAST thermocycler (Applied Biosystems). For quantification, a standard curve was included in each run with 10-fold serial dilutions of the target gene IS*1111*. The standard linear regression equation thus calculated was used to transform qPCR raw data from Cq values to an estimate of copy number per reaction tube. *C. burnetii* bacterial load was expressed as log transformed value of bacteria per gram, mililiter or swab, depending on the sample type, and was calculated taking into account the dilutions and volume transformations during sample processing and the target gene copy number in the Nine Mile reference strain (20 copies).

#### *C. burnetii* genotyping

Vaginal mucus, individual milk, air, slurry and dust samples, which had a positive qPCR result were submitted to Multiple Locus Variable number tandem-repeat Analysis (MLVA) to characterize the *C. burnetii* strains present in the herd along the 2 years. Two multicolor multiplex PCR assays were carried out targeting six microsatellite markers containing either six or seven base pairs (bp) repeat units: 3 hexanucleotide repeat markers (Ms27, Ms28 and Ms34) and 3 heptanucleotide repeat markers (Ms23, Ms24 and Ms33). Primer sequences used were as described before [[24],[25]]. The procedure has been described in detail elsewhere [[26]].

## Results

### Animals included in the study

At the beginning of the study, the herd size was 252 animals (177 cows, 45 heifers and 30 calves). Along the two years the census increased up to 289 animals with the incorporation of 165 heifers and the culling of 128 cows. Reproductive problems, mastitis or trauma were the main causes of culling. Therefore, a total of 392 animals were vaccinated along the study. Based on the pre-vaccination serostatus or PCR result, 311 of these animals were classified as being non-infected (98 cows, 22 heifers and 191 calves) while 81 were classified as infected (56 cows, 21 heifers and 4 calves).

### Progression of bacterial shedding

**Figure 1:**
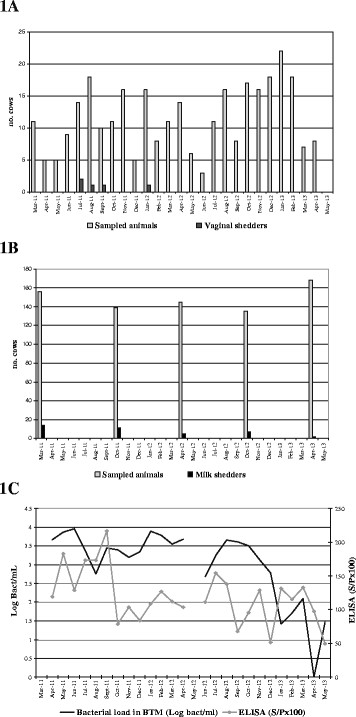
**Progression of** ***Coxiella burnetii*** **infection in the herd once vaccination started (April 2011). (A)** shedders through uterine fluids after calving; **(B)** shedders through milk among milking cows; **(C)***C. burnetii* load (log bact/ml) and ELISA titre of antibodies (S/Px100 ratio) against *C. burnetii* in bulk tank milk samples.

A total of 303 vaginal swabs collected from 217 cows post-calving were analyzed. Vaginal shedding was only detected from July to September 2011 and in January 2012 (Figure 1A). Five out of 217 cows shed *C. burnetii* DNA through uterine fluids (2.3%), 3 of them being first calving cows (3/104), a second calving cow (1/36) and one cow with more than 3 calvings (1/77). Quantification of the bacterial load present in the 5 positive samples showed that vaginal shedding was high (between 2.8 and 7.1 log bact/vaginal swab). Cows shedding *C. burnetii* through uterine fluids did not shed the organism again at the next calving.

Analysis of individual milk samples showed a gradual decline in the percentage of *C. burnetii* milk shedders throughout the study period (Figure 1B, Table 1). Before vaccination started in March 2011, 9.0% (14/156) of lactating cows were milk shedders. This prevalence was gradually reduced to 1.2% (2/168) in April 2013. The majority of *C. burnetii* milk shedders were cows with more than 3 parturitions (Table 1) and from April 2012 onwards, no shedders were detected among younger milking cows. The bacterial load of the positive milk samples varied between 1.4 and 5.6 log bact/ml milk. Along the study, 20 cows excreted *C. burnetii* DNA in one milking at least. Milk of 16 of these was analyzed more than one time. Eleven cows shed *C. burnetii* intermittently and the 5 shed *C. burnetii* persistently. The longest persistent excretion period recorded was 25 months (one cow). The reduced prevalence of milk shedder cows along the study caused a significant decline of *C. burnetii* bacterial burden in BTM at the end of the study (Figure 1C).

**Table 1:** Progression in the percentage of milk shedders after vaccination (April 2011) in the different age groups of milking cows in the different samplings

	**March 2011**		**October 2011**		**April 2012**		**October 2012**		**April 2013**	
	**N analyzed**	**% shedders**	**N analyzed**	**% shedders**	**N analyzed**	**% shedders**	**N analyzed**	**% shedders**	**N analyzed**	**% shedders**
1^st^ calving	46	4.3	45	2.2	44	0.0	44	0.0	67	0.0
2^nd^ calving	47	0.0	34	2.9	35	0.0	33	0.0	33	0.0
≥ 3 calvings	63	19.0	60	13.3	65	7.7	58	12.0	68	2.9
Total	156	9.0	139	7.2	144	3.5	135	5.2	168	1.2

Non-infected vaccinated animals were tested shortly after calving (i.e. approx. 10 months after vaccination) and none shed *C. burnetii* vaginally or through milk.

### Seroconversion rates in animals prior to vaccination

Sera from parturient cows, i.e. just before vaccination (n = 140) taken during the first study year were analyzed and the serostatus was compared with results from March 2011 to determine the seroconversion rate, i.e. seronegative to seropositive. Twelve out of the 140 cows seroconverted (8.6%). The highest rate of seroconversion was found among cows at their first (6/30) or second parturition (3/31), whereas cows with more than 3 calvings had a lower seroconversion rate (3/79). By April 2012 most of the cows in the herd had been vaccinated and seroconversion was therefore only investigated in 16 animals during the second study year. None of these seroconverted.

Kinetics of BTM antibodies are shown in Figure 1C. An initial increase of *C. burnetii* antibody levels was observed in BTM during the first six months of the study where more than half of the animals were vaccinated and the BTM antibody level reached a maximum in September 2011. After this, a decrease was observed and titers fluctuated slightly until the end of the study.

### Presence of *C. burnetii* DNA in the environment

**Figure 2:**
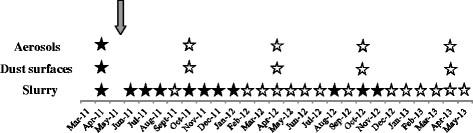
**Samplings and results on the presence of** ***Coxiella burnetii*** **DNA in environmental samples.** The arrow indicates the time when vaccination started (April 2011). Positive qPCR results are indicated with filled stars and negative results with open stars.

At the beginning of the study, slurry (1/1; 1.5 log bact/g), air samples (1/8; 2.7 log bact/ml) and dust from surfaces taken in the calving area (1/23; 1.9 log bact/swab) were positive for *C. burnetii* DNA (Figure 2). *C. burnetii* DNA was only detected in slurry samples after this time. In July 2011, August 2011 and January 2012, positive slurry samples with *C. burnetii* DNA levels of 2.5, 1.7 and 1.1 log bact/g, respectively, coincided with vaginal excretion in post-parturient cows (Figure 1A). However, slurry samples remained positive when vaginal excretion ceased in these cows. A total of 44% of the analyzed slurry samples were positive (11/25), the last positive being detected in November 2012 (Figure 2).

### Genotyping of *C. burnetii*

A selection of samples (n = 38), which were positive by qPCR with cycle threshold (Ct) values below 31 were genotyped by MLVA. These included uterine fluids (n = 9), individual milk (n = 25), BTM (n = 3) and slurry (n = 1). Genotype I [[26]] was identified in all samples. The genotype of the Nine Mile, RSA493 vaccine strain of *C. burnetii* was not identified.

## Discussion

Domestic ruminants are the main reservoir of *C. burnetii*. Infected animals shed the bacteria to the environment where they can persist for a long time [[10]] and create aerosols that expose humans and animals to the bacterium [[3]]. Vaccination against *C. burnetii* is considered a good option to prevent infection of ruminants [[13],[15]] and consequently exposure to humans of *C. burnetii*. Thus, recent studies confirmed the efficacy of vaccination in terms of preventing *C. burnetii* shedding in uninfected non-pregnant cows and calves [[11],[15]]. The study reported here, is the first to monitor the effectiveness of vaccination during two consecutive years in a dairy cattle farm naturally infected by *C. burnetii* regarding prevalence of animal shedders and contamination of the environment. Positive effects of vaccination were noticeable during the second year since seroconversions were not observed in susceptible animals thus indicating that horizontal transmission was no longer at a significant level. In addition to absence of seroconversion, vaccination also seemed to reduce vaginal excretion as uterine fluid samples were found negative by PCR during the last 15 study months. A low percentage of milk shedders and low bacterial load in BTM samples were still found at the end of the study. The reduced infections levels were reflected in all environmental samples being negative to *C. burnetii* in the last six months of the study period. Effectiveness of vaccination was associated with prevention of susceptible non-infected animals becoming *C. burnetii* shedders. In this sense, none of the uninfected vaccinated animals shed *C. burnetii* vaginally or through milk. This indicates an effective protection of phase I vaccine and confirmed the results of other authors who estimated that vaccinated, susceptible animals have five times less probability to become shedders than non-vaccinated animals [[15]].

An active culling strategy based on individual PCR results was not implemented in herd, but routine culling of animals due to common conditions such as reproductive problems, mastitis or trauma was performed. Although this was done without regard to *C. burnetii* status, culling of older cows and replacement with younger vaccinated heifers is expected to progressively have reduced *C. burnetii* infection prevalence, excretion rates and environmental contamination in the herd. The two year culling rate in the herd was 44% (128/289 cows). Thirteen out of the 20 milk shedders had been culled at the end of the study, including the five cows that were considered persistently milk shedders. It is not possible to differentiate the effects of vaccination *vs.* culling, but this study reflects the usual herd management procedures and in accordance with a previous study [[27]], it shows that vaccination and progressive culling of shedder cows is an effective method to reduce infection burden.

*C. burnetii* has previously been detected in aerosols of infected environments associated to small ruminants [[28],[29]] but this is the first study to assess the presence of *C. burnetii* in air and other environmental samples in a dairy cattle herd. Presence of *C. burnetii* in air, dust and slurry indicates the zoonotic risks associated with these materials. Calvings in a dairy cattle farm occur along the year, whereas in sheep flocks lambing is concentrated in a short period of time. As a result, after a Q fever episode, heavily infected sheep farms showed high environmental contamination at lambing [[19],[20]] with the consequent risks for uninfected animals and humans. In cattle, abortions due to *Coxiella* or bacterial shedding by infected animals happen intermittently along the year and the impact on air contamination with this zoonotic bacterium be consequently diluted.

Although preventive phase I vaccination reduces the risk of *C. burnetii* infection in uninfected animals [[15]], vaccination of infected herds does not have an immediate effect. In this sense, a recent study demonstrated that vaccination of already infected animals failed to reduce bacterial shedding [[30]]. The detection of positive environmental samples after almost two years of vaccination indicates that a long term vaccination and culling strategy is needed to reduce the potential for re-emergence of infection. This is in accordance with a study that estimated the effectiveness of different models of vaccination by using computer software and concluded that vaccination programs should be implemented during 10 years to be truly effective [[31]].

Genotyping of *C. burnetii* isolated from clinical and environmental samples has been helpful in identifying the strains involved in active Q fever episodes and to determine the ruminant sources involved in Q fever outbreaks [[25],[26],[32]]. In the current study, *C. burnetii* genotype I was identified in all samples. This genotype has previously been isolated in bovine milk in several European countries such as France, Holland, Portugal and Switzerland [[33]] and it has also been found in clinical samples of human placenta and heart valve in France between 1994 and 1996 [[34]]. This indicates the potential role of cattle in the domestic cycle of *C. burnetii* and the importance of implementing efficient farm-based control measures.

## Conclusions

The overall reduction of *C. burnetii* infection in animals to 1.2% milk shedders and the reduced environment contamination is ascribed to the effects of vaccination together with the culling of milk shedders. Vaccination has to be planned as a medium-long term strategy to suppress risks of re-infection.

## Abbreviations

BTM: Bulk tank milk

EFSA: European Food Safety Authority

RD: Real Decreto (Spanish) or Spanish Royal Decree law

PCR: Polymerase chain reaction

qPCR: Quantitative real-time PCR

MLVA: Multiple locus variable number tandem repeat analysis

## Competing interests

The authors declare that they have no competing interests.

## Authors’ contributions

AP was responsible for laboratorial analyses, assisted with interpretation of data and drafted of the manuscript; JB carried out the field work and samplings, and assisted with discussion of results; AH supervised molecular laboratory work, and critically revised the manuscript; AG conceived the study, designed the sampling protocol and wrote the manuscript. All authors revised the manuscript and approved it in its final version.

## Ackowledgements

This study was supported by Spanish National Institute for Agricultural and Food Research and Technology (INIA, RTA 2009-00017-00) and the European Regional Development Fund (ERDF). AP is the recipient of a predoctoral fellowship from INIA.

We acknowledge the interest and collaboration of the farmer and the veterinarian (D. García).
